# Impact of extracorporeal membrane oxygenation treatments on acquired von Willebrand syndrome in patients with out-of-hospital cardiac arrest: a retrospective observational study

**DOI:** 10.1186/s12959-024-00617-4

**Published:** 2024-05-31

**Authors:** Yuki Chiba, Kota Goto, Misako Suzuki, Hisanori Horiuchi, Mineji Hayakawa

**Affiliations:** 1 Division of Medical Engineering Center, Hokkaido University Hospital, Sapporo, Japan; 2 Department of Molecular and Cellular Biology, Institute of Development, Aging and Cancer, Tohoku University, Sendai, Japan; 3 Emergency and Critical Care Center, Hokkaido University Hospital, Hokkaido University Hospital, N14W5 Kita-ku, Sapporo, 060-8648 Japan

**Keywords:** Cardiopulmonary resuscitation, Extracorporeal membrane oxygenation, Heart arrest, Von Willebrand factor, Out-of-hospital cardiac arrest

## Abstract

**Background:**

Von Willebrand factor (vWF) plays a crucial role in hemostasis, acting as a key factor for platelet adhesion/aggregation and as a transport protein for coagulation factor VIII. vWF is secreted as a giant multimer, and it undergoes shear stress-dependent cleavage by a specific metalloproteinase in plasma. Among vWF multimers, high-molecular-weight (large) multimers are essential for hemostasis. Acquired von Willebrand syndrome, linked to various conditions, is a hemostatic disorder due to reduced vWF activity. Extracorporeal membrane oxygenation (ECMO), utilized recently for out-of-hospital cardiac arrest patients, generates high shear stress inside the pump. This stress may induce a conformational change in vWF, enhancing cleavage by a specific metalloproteinase and thereby reducing vWF activity. However, no study has investigated the effects of ECMO on vWF-related factors in patients receiving or not receiving ECMO. This study aimed to elucidate the relationship between ECMO treatment and acquired von Willebrand syndrome-related factors in patients with out-of-hospital cardiac arrest.

**Methods:**

This study included patients with cardiogenic out-of-hospital cardiac arrest admitted to our hospital. The patients were categorized into two groups (ECMO and non-ECMO) based on the presence or absence of ECMO treatment. Plasma samples were collected from patients admitted to the emergency department (days 0–4). The vWF antigen (vWF: Ag), vWF ristocetin cofactor activity (vWF: RCo), and factor VIII activity were measured. Additionally, a large multimer of vWF was evaluated through vWF multimer analysis, utilizing western blotting to probe vWF under non-reducing conditions.

**Results:**

The ECMO and non-ECMO groups included 10 and 22 patients, respectively. The median ECMO treatment in the ECMO group was 64.6 h. No differences in vWF: Ag or factor VIII activity were observed between the two groups during the observation period. However, the ECMO group exhibited a decrease in large vWF multimers and vWF: RCo during ECMO. Strong correlations were observed between vWF: RCo and vWF: Ag in both groups, although the relationships were significantly different between the two groups.

**Conclusions:**

ECMO treatment in patients with out-of-hospital cardiac arrest resulted in the loss of large vWF multimers and decreased vWF activity. Hence, decreased vWF activity should be considered as a cause of bleeding during ECMO management.

**Supplementary Information:**

The online version contains supplementary material available at 10.1186/s12959-024-00617-4.

## Background

Public awareness of extracorporeal membrane oxygenation (ECMO) treatments has increased owing to their use in treating patients with severe coronavirus disease 2019 [1–3]. There are two common ECMO treatment methods: venovenous ECMO (VV-ECMO) for respiratory support and venoarterial ECMO (VA-ECMO) for circulatory support. VA-ECMO serves as a temporary but robust mechanical support option [4]. Additionally, extracorporeal cardiopulmonary resuscitation (E-CPR) involving ECMO has been recommended for patients with refractory ventricular fibrillation (VF), leading to out-of-hospital cardiac arrest (OHCA) [5]. However, bleeding complications occur in approximately 30–50% of these ECMO treatments, posing a significant issue [6, 7].

Von Willebrand factor (vWF) is a crucial component of the hemostatic process, serving as a key factor in platelet adhesion/aggregation and as a transport protein for coagulation factor VIII [8]. vWF is produced and secreted as a giant multimer, cleaved by a metalloproteinase, disintegrin-like, and metalloproteinase with thrombospondin type 1 motif 13 (ADMTS13), in a shear stress-dependent manner. It is present in plasma as multimers comprising 2–80 vWF subunits, with high molecular weight (large) multimers being essential for hemostasis [8, 9].

Although a congenital abnormality of vWF causes a hemostatic disorder that is recognized as von Willebrand disease [10], various conditions induce an acquired abnormality of vWF activity, known as acquired von Willebrand syndrome [9]. Certain diseases, such as autoimmune diseases, malignancies, and cardiovascular diseases with unphysiologically high shear stress, such as aortic stenosis, are recognized causes of acquired von Willebrand syndrome. Among these, cardiovascular disease is the most frequent, where high shear stress in the bloodstream leads to excessive degradation of vWF multimers, resulting in the loss of large vWF multimers [9].

Several reports have indicated the relationship between ECMO and acquired von Willebrand syndrome. One noted a decrease in the ratio of the collagen-binding capacity of vWF to the amount of vWF antigen from before to after the start of VV-ECMO treatment [11]. Another found that large vWF multimers in five patients with VA-ECMO for circulatory shock were significantly lower than those in patients with severe aortic stenosis (Heyde syndrome) [12]. However, no comparative studies between patients with and without ECMO treatment exist, and there is a lack of studies indicating a relationship between acquired von Willebrand syndrome and E-CPR. In this study, we aimed to elucidate the relationship between ECMO treatment and acquired von Willebrand syndrome-related factors in patients with OHCA.

## Materials & methods

### Setting

This study was approved by the Institutional Review Boards of the Ethics Committees of Hokkaido University Hospital (No. 022–0122, approved December 28, 2022) and Tohoku University School of Medicine (No. 27,919, March 16, 2023) and conducted in accordance with the Helsinki Declaration. The need for additional written informed consent was waived because of the retrospective study design involving the use of samples already in storage.

A single-center retrospective observational study was conducted by enrolling patients with OHCA admitted to the Emergency and Critical Care Center at Hokkaido University Hospital between September 2019 and January 2023. Exclusion criteria were as follows: non-cardiac etiology, no witnessed cardiac arrest, age < 18 years, and insufficient data. Blood samples were prospectively collected daily from arrival at the emergency department (day 0) to day 4. Written informed consent was obtained from the patient or his/her next of kin by comprehensive consent. The blood sample was collected in a 3.2% sodium citrate-containing tube and separated through serial centrifugation (15 min at 3,500 rpm at 25 °C, twice). The supernatant was collected and frozen at − 80 °C until analysis.

All patients received standard cardiac resuscitation and postcardiac arrest care according to standard protocols. Moreover, some patients were resuscitated using ECMO, whereas others were not. The criteria for introduction of ECMO were as follows: (1) ventricular fibrillation or pulseless ventricular tachycardia on the initial electrocardiogram, (2) cardiac arrest on hospital arrival with or without pre-hospital return of spontaneous circulation (ROSC), (3) interval of < 45 min between reception of the emergency call or the onset of cardiac arrest and hospital arrival, and (4) age < 75 years. The final decision to introduce ECMO was made by the attending physician. Additional emergency coronary interventions, including coronary angiography and percutaneous coronary intervention, were conducted for patients with suspected acute coronary syndrome. To assess the effect of ECMO treatment on vWF-related values, patients were divided into two groups based on the presence or absence of ECMO treatment: ECMO and non-ECMO groups.

### Sample measurements and data collection

vWF ristocetin cofactor activity (vWF: RCo) (BC Von Willebrand Reagent®, Siemens Healthcare Diagnostics, Marburg, Germany) [13], vWF antigen (vWF: Ag) (vWF Ag reagent®, Siemens Healthcare Diagnostics) [14], and activity of coagulation factor VIII (FVIII) (RevohemTM FVIII Chromogenic, Sysmex, Kobe, Japan) were measured using an automated coagulation analyzer, CS-6000™ (Sysmex, Kobe, Japan), following the manufacturer’s instructions.

vWF multimer analysis utilized a 1.0% agarose gel, where an equal amount of vWF: Ag was analyzed with western blotting under non-reducing conditions using an anti-vWF antibody (DAKO, Glostrup, Denmark) as the primary antibody. Quantitative evaluation of large vWF multimers was done using a large vWF multimer index [15–17]. The western blotting results classified the vWF multimer bands as small multimers from the 5th to the bottom, medium multimers from the 6th to the 10th, and large multimers from the 11th onward. The bands of the vWF multimer in the patient and control (Siemens Standard plasma) were simultaneously analyzed using densitometry (ImageJ, NIH, United States). The large multimer bands were divided by the corresponding total vWF bands, and the value was defined as the vWF large multimer ratio. Lastly, the vWF large multimer index was calculated as the ratio of the patient’s vWF large multimer ratio to the control’s vWF large multimer ratio.

Clinical information and conventional laboratory results were collected from the medical records and emergency service reports.

### Statistics analysis

All measurements are expressed as median (interquartile range) or number (percentage). Continuous and categorical variables were compared between groups using the Wilcoxon signed-rank test and the chi-square test, respectively. Regression analysis was performed to determine the relationship between variables in each group. Additionally, an analysis of covariance (ANCOVA) was used to investigate the differences in the relationship between variables across groups. Pearson’s correlation coefficients were calculated to determine correlations between two variables. Statistical significance was defined as *P* < 0.05, and the Bonferroni correction was used when necessary. Statistical analyses were performed using JMP version 16.2.0 (SAS Institute Inc., Cary, NC, USA).

## Results

This study evaluated 32 patients from a total of 80 patients with OHCA, excluding 29 patients with non-cardiac etiology, nine patients without witnessed cardiac arrest, one patient aged < 18 years, and nine patients with missing data. Of the 32 patients, 10 and 22 were assigned to the ECMO and non-ECMO groups, respectively.

Table 1 summarizes the baseline characteristics of the study participants. The patients in the ECMO group were younger than those in the non-ECMO group. The CPR duration (no- and low-flow times) in the ECMO group was longer than that in the non-ECMO group; the ECMO group received a higher dose of adrenaline than the non-ECMO group, reflecting this longer CPR time. Despite the influence of ABO blood type on plasma levels of vWF: Ag [18], the ABO blood types were not different between groups. Laboratory data upon arrival at the hospital are presented in Table 2. In the ECMO group, two patients’ blood samples were collected prior to ECMO treatment, whereas those from the other patients were collected after the start of ECMO treatment. Platelet count and various coagulation variables were worse in the ECMO group than in the non-ECMO group. Table 3 presents the information on ECMO treatments.

**Table 1:** Characteristics of patients

	All patients (*n* = 32)	ECMO (*n* = 10)	Non–ECMO (*n* = 22)	*P*–value
Age, years	60 (27–91)	49 (41–73)	67 (27–91)	0.019
Male sex (n, %)	27 (84.4)	9 (90.0)	18 (81.8)	0.541
Body mass index*	24.2 (16.6–30.1)	25.4 (20.1–30.1)	23.7 (16.6–29.7)	0.183
Bystander CPR (n, %)	25 (78.1)	6 (60.0)	19 (86.4)	0.105
Defibrillation before arrival at ER (n, %)	25 (78.1)	8 (80.0)	17 (77.3)	0.862
CPR duration				
No flow time (min)	0 (0–16)	6 (0–16)	0 (0–15)	0.005
Low flow time (min)	30 (5–61)	50.5 (32–61)	26 (5–50)	< 0.001
Initial rhythm (n, %)				
Asystole	4 (12.5)	0 (0)	4 (18.2)	0.195
PEA	6 (18.8)	2 (20.0)	4 (18.2)	
VF/VT	21 (65.6)	8 (80.0)	14 (63.6)	
Total adrenaline use (mg)	1 (0–8)	3.5 (0–7)	1 (0–8)	0.046
Blood types (n, %)				
A	13 (40.6)	3 (30.0)	10 (45.5)	0.282
B	4 (12.5)	3 (30.0)	1 (4.5)	
O	11 (34.4)	3 (30.0)	8 (36.4)	
AB	4 (12.5)	1 (10.0)	3 (13.6)	

* No data are available for one patient. CPR, cardiopulmonary resuscitation; PEA, pulseless electrical activity; VF, ventricular fibrillation; VT, ventricular tachycardia

**Table 2:** Laboratory data on arrival at the hospital

	Reference value	All patients (*n* = 32)	ECMO (*n* = 10^*^)	Non–ECMO (*n* = 22)	*P*–value
WBC, ×10^9^/L	3.3–8.6	10.7 (7.0–19.1)	10.7 (7.0–17.9)	17.9 (8.6–19.1)	0.180
Hemoglobin, g/L	137–168	132 (121–150)	129 (121–150)	136 (122–139)	0.714
Platelet, ×10^9^/L	158–348	168 (117–263)	155 (117–258)	256 (156–263)	0.027
PT–INR	0.86–1.08	1.2 (1.1–1.9)	1.3 (1.1–1.9)	1.1 (1.1–1.4)	0.004
APTT, sec	25.4–36.4	39.4 (30.1–69.2)	40.9 (30.1–69.2)	32.9 (30.2–46.3)	0.014
Fibrinogen, g/L	2.00–4.00	2.47 (1.39–3.98)	2.45 (1.47–3.98)	2.47 (1.39–3.45)	0.056
Antithrombin, %	80–130	78 (53–93)	64 (53–90)	86 (78–93)	0.046
FDP, µg/mL	< 5	47.1 (5.7–161.3)	47.1 (5.7–104.0)	152.9 (14.7–161.3)	0.264
D–dimer, µg/mL	< 1	18.1 (1.0–37.1)	18.3 (1.0–32.0)	18.1 (2.3–37.1)	0.882
SFMC, µg/mL	< 7	49.2 (11.8–211.0)	45.8 (32.0–170.0)	52.6 (11.8–211.0)	0.577
Blood gas analysis					
pH	7.36–7.46	7.1 (6.6–7.4)	7.0 (6.7–7.3)	7.1 (6.6–7.4)	0.215
Lactate, mmol/L	0.5–1.6	10.9 (2.3–23.0)	14.5 (6.1–21.0)	9.6 (2.3–23.0)	0.017

* Eight patients in the ECMO group after ECMO treatment and two before ECMO treatment. WBC, white blood cell; PT–INR, prothrombin time–international normalized ratio; APTT, activated partial thromboplastin time; FDP, fibrinogen degradation product; SFMC, soluble fibrin monomer complex

**Table 3:** Information on Extracorporeal membrane oxygenation treatments

	*n* = 10
Arterial cannula 15/16/18 Fr, n	3 / 4 / 3
Venous cannula 21/22/24 Fr, n	3 / 5 / 2
Centrifugal pump speed, rpm	
At the time of ECMO introduction, rpm	3485 (3000–4000)
Average during ECMO treatment, rpm	3054 (2555–3521)
Duration of ECMO treatment, hours	64.6 (26.2–258.4)

ECMO, extracorporeal membrane oxygenation

**Fig. 1:**
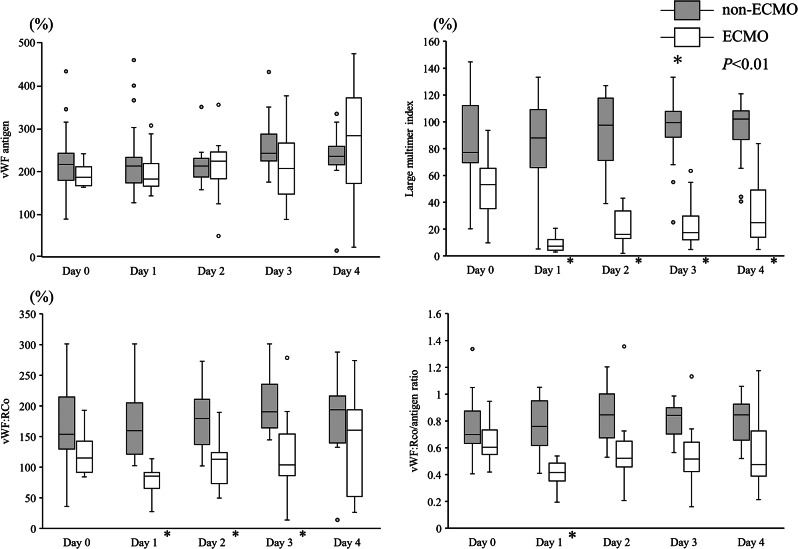
Von Willebrand factor-related measurements in ECMO and non-ECMO groups. Blood samples were collected from day 0 (upon arrival at the emergency department) to day 4. The von Willebrand factor (vWF) antigen levels did not differ between the ECMO and non-ECMO groups. The vWF ristocetin cofactor activities (vWF: Rco) and vWF: Rco/antigen ratios were significantly different between these groups. Large multimeric indices were also clearly different between these groups. ECMO, Extracorporeal membrane oxygenation; vWF, von Willebrand factor; vWF: Rco, vWF ristocetin cofactor activity; white box, ECMO group; gray box, non-ECMO group; * *P* < 0.01 after Bonferroni correction (Initial *P*-value < 0.05)

Figure 1 illustrates vWF: Ag, vWF: RCo, the vWF: RCo/vWF: Ag ratio, and the vWF large multimer index in the ECMO and non-ECMO groups. No significant differences were found in vWF: Ag between the ECMO and non-ECMO groups during the 5 days after arrival at the emergency department. However, vWF: RCo was significantly lower in the ECMO group than in the non-ECMO group from day 1 to day 3 (*P* < 0.01). The vWF: RCo/vWF: Ag ratio in the ECMO group was lower than that in the non-ECMO group on day 1 (*P* < 0.01). The vWF large multimer index was lower in the ECMO group than in the non-ECMO group, except on admission (day 0) (*P* < 0.01).

**Fig. 2:**
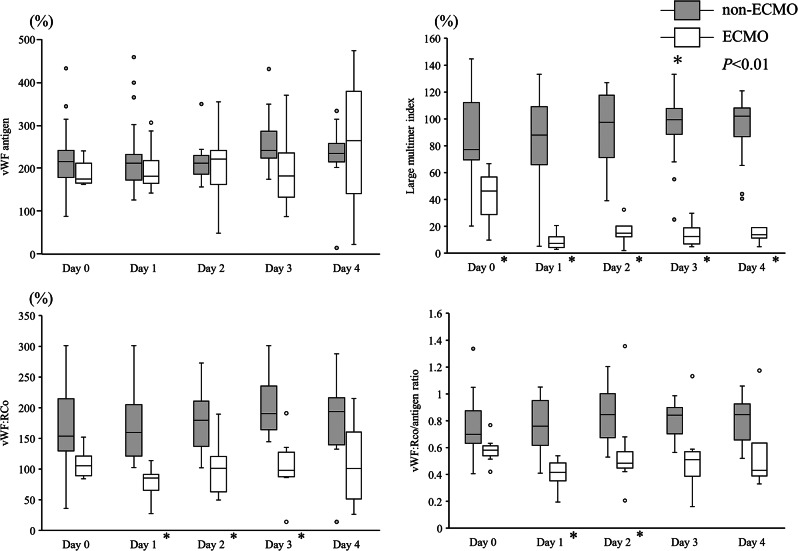
Von Willebrand factor-related measurements in patients during ECMO treatments in ECMO and non-ECMO groups. In the ECMO group, the results of the patients before and after ECMO were excluded. The number of patients in the ECMO group was 8 on day 0, 10 on day 1, 8 on day 2, 6 on day 3, and 4 on day 4. The von Willebrand factor (vWF) antigen levels did not differ between the ECMO and non-ECMO groups. The vWF ristocetin cofactor activities (vWF: Rco) and vWF: Rco/antigen ratios were significantly different between these groups. Large multimeric indices were also clearly different between these groups. ECMO, Extracorporeal membrane oxygenation; vWF, von Willebrand factor; vWF: Rco, vWF ristocetin cofactor activity; white box, ECMO group; gray box, non-ECMO group; * *P* < 0.01 after Bonferroni correction (Initial *P*-value < 0.05)

In the subgroup analysis, Fig. 2 presents only the results of samples treated with ECMO in the ECMO group. In other words, we excluded the results of samples from the ECMO group before the start of ECMO treatment on day 0 and the results of samples after the end of ECMO treatment between days 2 and 4. The numbers of patients in the ECMO group were 8, 10, 8, 6, and 4 on days 0, 1, 2, 3, and 4, respectively. Differences in the vWF: RCo/vWF: Ag ratio and vWF large multimer index between the ECMO and non-ECMO groups are more apparent in Fig. 2 than in Fig. 1.

**Fig. 3:**
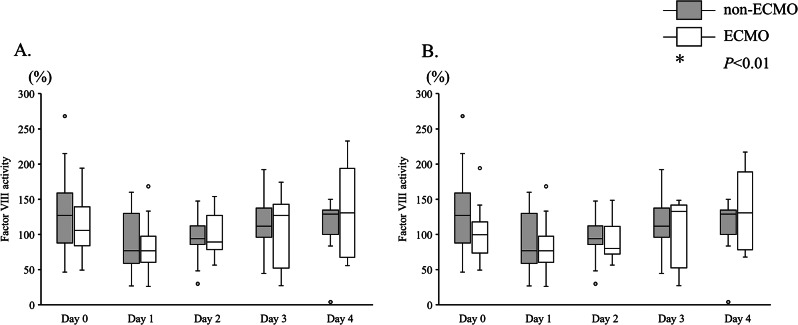
Coagulation factor VIII activity in the ECMO and non-ECMO groups. **A**: Coagulation factor VIII activity measurements in the ECMO and non-ECMO groups. Coagulation factor VIII activity did not differ between the ECMO and non-ECMO groups. **B**: Coagulation factor VIII activity measurements during ECMO treatment in the ECMO and non-ECMO groups. In the ECMO group, the results for patients before and after ECMO were excluded. The number of patients in the ECMO group was 8 on day 0, 10 on day 1, 8 on day 2, 6 on day 3, and 4 on day 4. Coagulation factor VIII activity did not differ between the ECMO and non-ECMO groups. ECMO, Extracorporeal membrane oxygenation; White box, ECMO group; gray box, non-ECMO group. * *P* < 0.01 after Bonferroni correction (initial *P*-value of < 0.05)

There were no significant differences in FVIII activity between the ECMO and non-ECMO groups during the 5 days after arrival at the emergency department (Fig. 3).

**Fig. 4:**
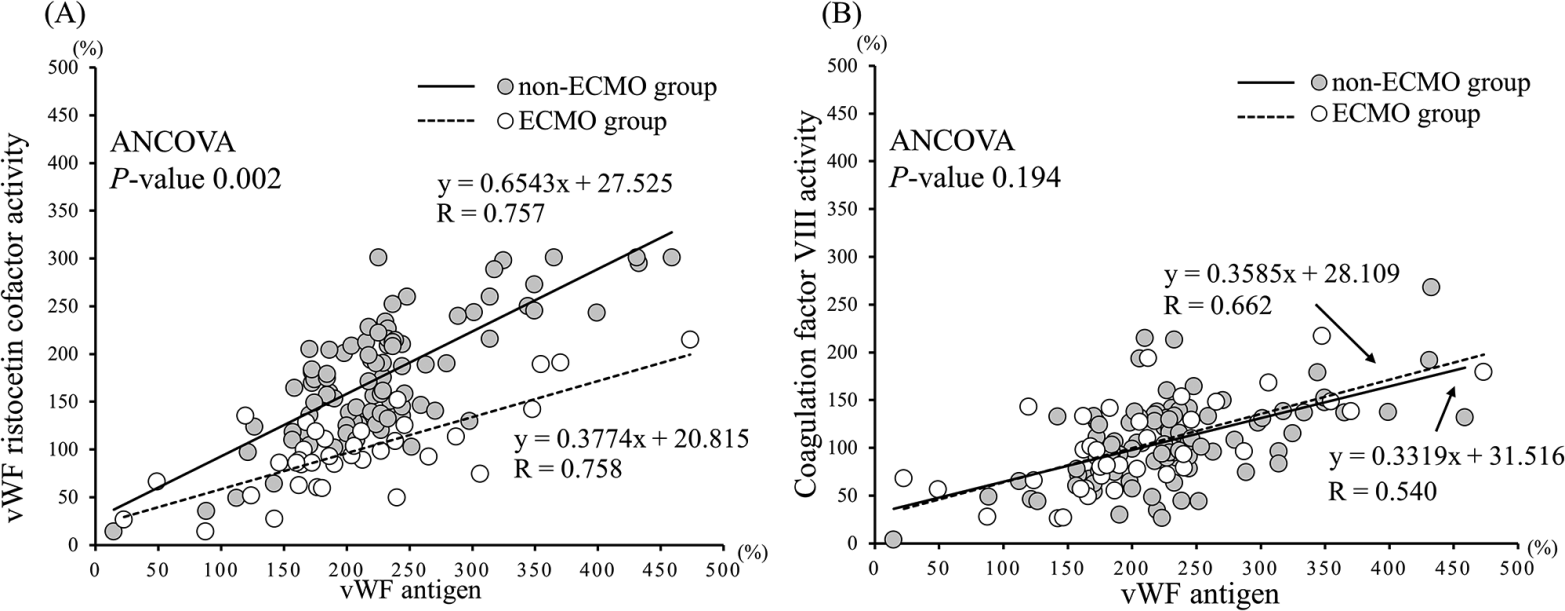
Relationship between Von Willebrand factor-related measurements in samples with and without ECMO treatment. In the ECMO group, results from specimens collected in the absence of ECMO treatment (before the start of ECMO support and after discontinuing ECMO treatment) were excluded. White circles, ECMO group; grey circles, non-ECMO group; dotted line, regression line in the ECMO group; solid lines, regression line in the non-ECMO group. **A**. Relationship between von Willebrand factor antigen and ristocetin cofactor activity. The relationship between the von Willebrand factor antigen and ristocetin cofactor activity was significantly different between the ECMO and non-ECMO groups (*P* = 0.002 by analysis of covariance). **B**. Relationship between the von Willebrand factor antigen and coagulation factor VIII activity. The relationship between the von Willebrand factor antigen and coagulation factor VIII activity was not significantly different between the ECMO and non-ECMO groups. ECMO, Extracorporeal membrane oxygenation; vWF, von Willebrand factor; ANCOVA, analysis of covariance

**Fig. 5:**
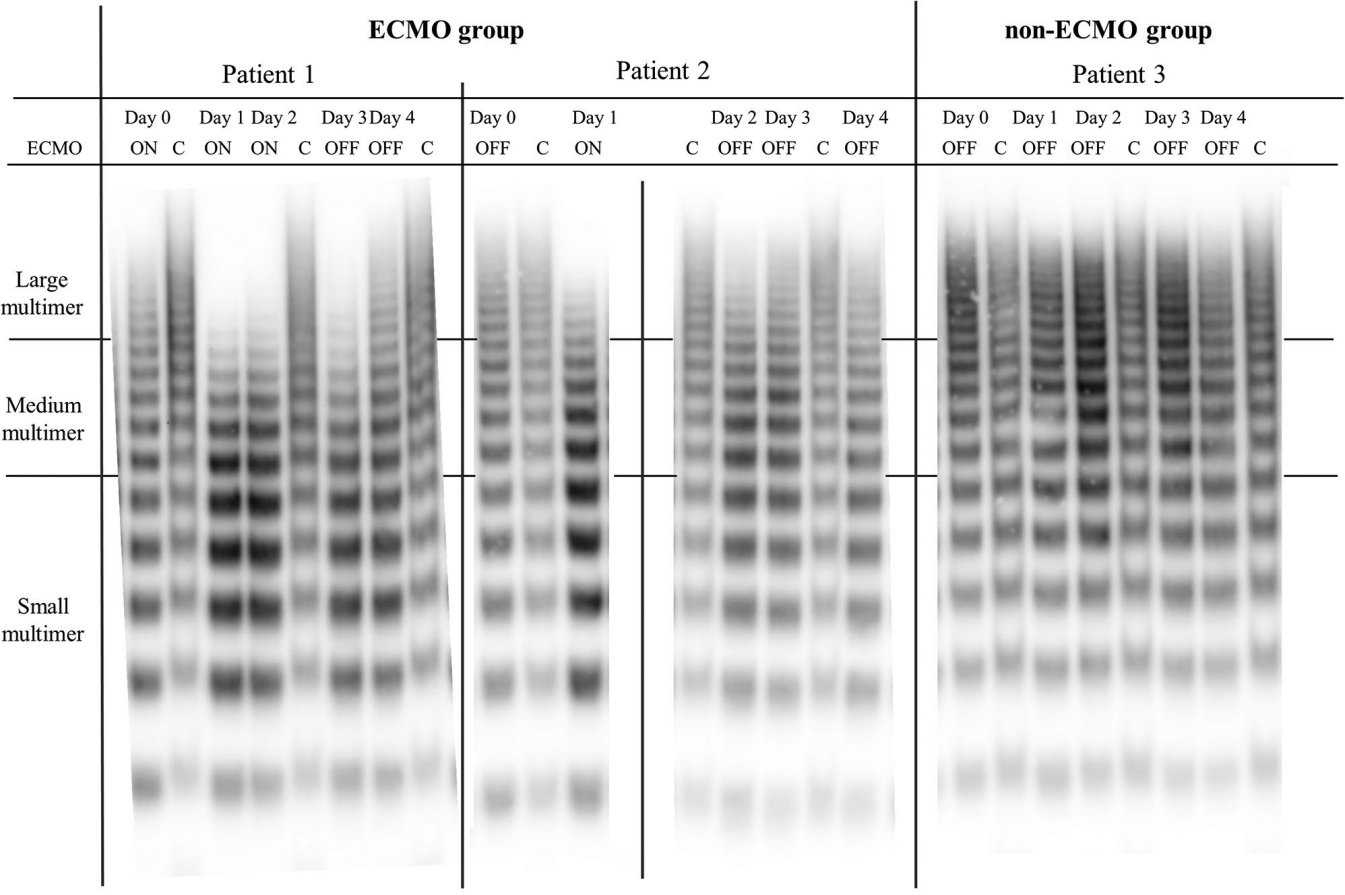
Representatives of western blot of von Willebrand factor multimer in ECMO and non-ECMO groups. In patients 1 and 2 (ECMO group), the large multimers immediately disappeared after starting ECMO treatment and were gradually restored after terminating ECMO treatment. However, in patient 3 (non-ECMO group), the large multimers did not disappear during the observation period. ECMO, Extracorporeal membrane oxygenation; ON, sample collected during ECMO; OFF, sample collected without ECMO; C, healthy controls

Figure 4A illustrates the relationship between vWF: RCo and vWF: Ag in patients receiving or not receiving ECMO. In the ECMO group, results from specimens collected in the absence of ECMO treatment (before the start of ECMO support and after the discontinuation of ECMO treatment) were excluded. Despite strong correlations between vWF: RCo and vWF: Ag in both groups (*r* = 0.757 in the ECMO group and *r* = 0.758 in the non-ECMO group), the relationships were statistically different (*P* = 0.002 by ANCOVA). Figure 4B displays the relationship between FVIII activity and vWF: Ag in the ECMO and non-ECMO samples. Although moderate correlations between vWF: RCo and vWF: Ag were observed in both groups, these relationships were not significantly different. Representative results of the vWF multimer analysis using western blotting are shown in Fig. 5. The disappearance of the large vWF multimer was observed in samples treated with ECMO. Additionally, recovery of the vWF large multimer index was observed after ECMO.

**Fig. 6:**
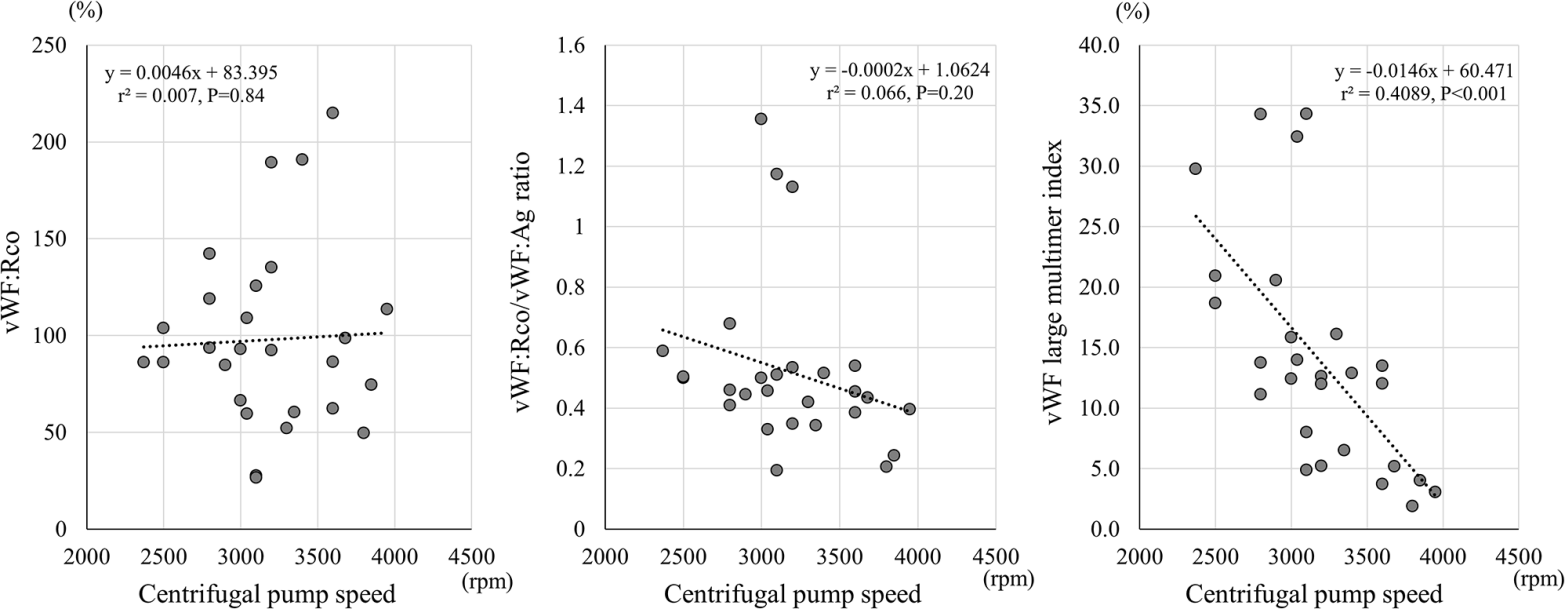
Correlations of Von Willebrand factor-related measurements with the centrifugal pump speed during ECMO treatment. The graphs (left to right) illustrate the correlations between the centrifugal pump speed in ECMO and vWF: RCo, the vWF: RCo/vWF: Ag ratio, and the vWF large multimer index, respectively. However, the results on day 0 are excluded because they were recorded just after the introduction of ECMO. The vWF large multimer index decreased with an increasing in the centrifugal pump speed (r²=0.4089, *P* < 0.001). vWF, von Willebrand factor; vWF: Rco, vWF ristocetin cofactor activity; rpm, rotations per minute

Figure 6 illustrates the relationship between the centrifugal pump speed in ECMO and vWF: RCo, the vWF: RCo/vWF: Ag ratio, and the vWF large multimer index. Although the centrifugal pump speed showed no correlation with vWF: Rco and the vWF: RCo/vWF: Ag ratio, it showed a strong correlation with the vWF large multimer index (r^2^ = 0.4089, *P* < 0.001).

## Discussion

This study aimed to evaluate the effects of ECMO on acquired von Willebrand syndrome in patients with OHCA. Although the vWF: Ag levels did not differ between the ECMO and non-ECMO groups, the vWF: RCo levels and vWF: RCo/vWF: Ag ratio were significantly lower in the ECMO group than in the non-ECMO group. Furthermore, the vWF large multimer index was lower in the ECMO group than in the non-ECMO group.

To the best of our knowledge, this is the first report comparing acquired von Willebrand syndrome-related factors between groups, with and without ECMO treatment. Various stimuli release vWF from endothelial cells, and vWF: Ag levels are significantly altered [19]. Although the standard value of vWF: Ag is 50–155%, the median vWF: Ag levels were approximately 200% during the observation period in the non-ECMO group (control group) in this study. Hence, it is essential to compare the changes in vWF-related factors in patients receiving ECMO with those in patients with similar underlying diseases who are not receiving ECMO (non-ECMO group) to clarify the changes in vWF-related factors in patients receiving ECMO.

Although the vWF: Ag levels did not differ between the ECMO and non-ECMO groups, the vWF: RCo levels in the non-ECMO group were higher than those in the ECMO group. Likewise, the vWF: Rco/vWF: Ag ratio decreased during ECMO treatment, unlike the non-ECMO group. In the ECMO group, vWF underwent conformational changes due to the shear stress of the centrifugal pump of ECMO and increased degradation by ADAMTS-13 [20, 21]. This phenomenon is supported by the finding of a decrease in the vWF large multimer index with an increase in the centrifugal pump speed (Fig. 6). Therefore, the large vWF multimer was cleaved to form a small multimer, decreasing its activity. Previous studies revealed relationships between acquired von Willebrand syndrome and ECMO treatment [22, 23]. However, these studies compared the vWF-related factors before and after ECMO treatments in patients [22, 23]. This is the first study to compare the acquired von Willebrand syndrome-related factors over time in patients with and without ECMO treatment.

The vWF large multimer index showed a more clear difference between groups than did vWF: Ag and the vWF: Rco/vWF: Ag ratio. Furthermore, a strong correlation was clearly observed between the vWF large multimer index and the centrifugal pump speed. Considering the pathophysiology of ECMO-related acquired von Willebrand syndrome wherein large vWF multimers are cleaved into small multimers, we believe that a large multimer index can reflect its pathophysiology. To evaluate the large multimer index, western blotting is needed. Hence, evaluating large multimer indices remains challenging in a clinical setting.

As observed for the patients in the ECMO group in this study, the loss of large vWF multimers is one of the hallmarks of von Willebrand disease type 2 A [10]. However, several characteristics of patients in the ECMO group differed from those of patients with von Willebrand disease type 2 A. Usually, patients with von Willebrand disease type 2 A show low vWF; Ag, vWF: Rco < 30 IU/dL (30%), and vWF: Rco/vWF: Ag ratio < 0.6 [10]. In the ECMO group in this study, the vWF: Rco/vWF: Ag ratio was < 0.6; however, vWF: Ag was almost double of that observed in healthy subjects. vWF is released from the endothelial cells on various types of stimulation, with an increase in vWF: Ag levels [8]. In the present study, endothelial cell stimulation by cardiac arrest and resuscitation induced the release of vWF and increased vWF: Ag levels [8, 24]. However, the association between these changes in vWF and bleeding complications was not evaluated.

In two recent prospective observational studies of neonates and children receiving ECMO, acquired von Willebrand syndrome was inevitable during the treatment, similar to the findings in the present study; however, there was no association between major bleeding and vWF-related variables [25, 26]. Various risk factors for bleeding exist during ECMO treatment, including placement of large cannulae, administration of anticoagulant/antiplatelet medications, and abnormal hemostasis due to serious underlying disease [6, 7]. Therefore, given the multifactorial etiologies of bleeding complications, it would be very difficult to assess the direct impact of acquired von Willebrand syndrome on such complications during ECMO treatment.

In this study, although vWF activity and vWF multimer size were affected by ECMO, factor VIII activity did not differ between groups during the observation period. vWF protects factor VIII from degradation by activated protein C via noncovalent binding [10, 27]. The binding site for factor VIII is the D’-D3 domain in a vWF monomer [10, 27]. Patients with congenital von Willebrand’s disease (types 1 and 3) with reduced vWF: Ag levels also have reduced factor VIII activity [10]. However, Bansal et al. reported that left ventricular assist device-induced acquired von Willebrand syndrome presented with decreased vWF activity but did not reduce factor VIII activity [28], similar to the results of the present study. In ECMO-induced acquired von Willebrand syndrome, large vWF multimers are cleaved into small multimers; however, vWF: Ag levels did not decrease. Furthermore, small vWF multimers have factor VIII binding capacity similar to that of a large multimer [29–31]. Therefore, in the present study, factor VIII activity in the ECMO group did not decrease, and the relationships between vWF: Ag levels and factor VIII did not differ between the ECMO and non-ECMO groups. These findings were similar to those of studies comparing patients with aortic stenosis patients with a control group [31].

This study had several limitations. First, vWF is an important factor related to hemostasis; however, information on bleeding status was not obtained. The risk of bleeding significantly differed between the patients treated with and without ECMO. Furthermore, we could not distinguish between acquired von Willebrand syndrome–related bleeding and other bleeding types. Second, the study was a single-center study, with a small number of patients and an underpowered design. Third, shear stress on vWF was anticipated to increase as the ECMO rotation speed increased; however, owing to the different backgrounds of each patient, observing a relationship between ECMO rotation speed and changes in vWF was not feasible.

## Conclusions

ECMO treatment in patients with OHCA causes vWF fragmentation, leading to the loss of large vWF multimers and decreased vWF activity. Hence, decreased vWF activity should be considered a cause of bleeding during ECMO management.

## Electronic supplementary material

Below is the link to the electronic supplementary material.

Supplementary Material 1

Supplementary Material 2

## Data Availability

The data supporting the findings of this study are available from the corresponding author, M Hayakawa, on reasonable request.

## Abbreviations

vWF: Von Willebrand factor
ECMO: Extracorporeal membrane oxygenation
vWF: Ag: vWF antigen
vWF: RCo: vWF ristocetin cofactor activity
VA-ECMO: Venoarterial ECMO
E-CPR: Extracorporeal cardiopulmonary resuscitation
VF: Ventricular fibrillation
OHCA: Out-of-hospital cardiac arrest
ADMTS13: Metalloproteinase with thrombospondin type 1 motif 13
FVIII: Factor VIII
ANCOVA: Analysis of covariance
