# Supplementary figures and images for: Impact of extracorporeal membrane oxygenation treatments on acquired von Willebrand syndrome in patients with out-of-hospital cardiac arrest: a retrospective observational study
Source: Thromb J. 2024 May 31;22:46. doi: 10.1186/s12959-024-00617-4 (PMC11143620; doi:10.1186/s12959-024-00617-4)

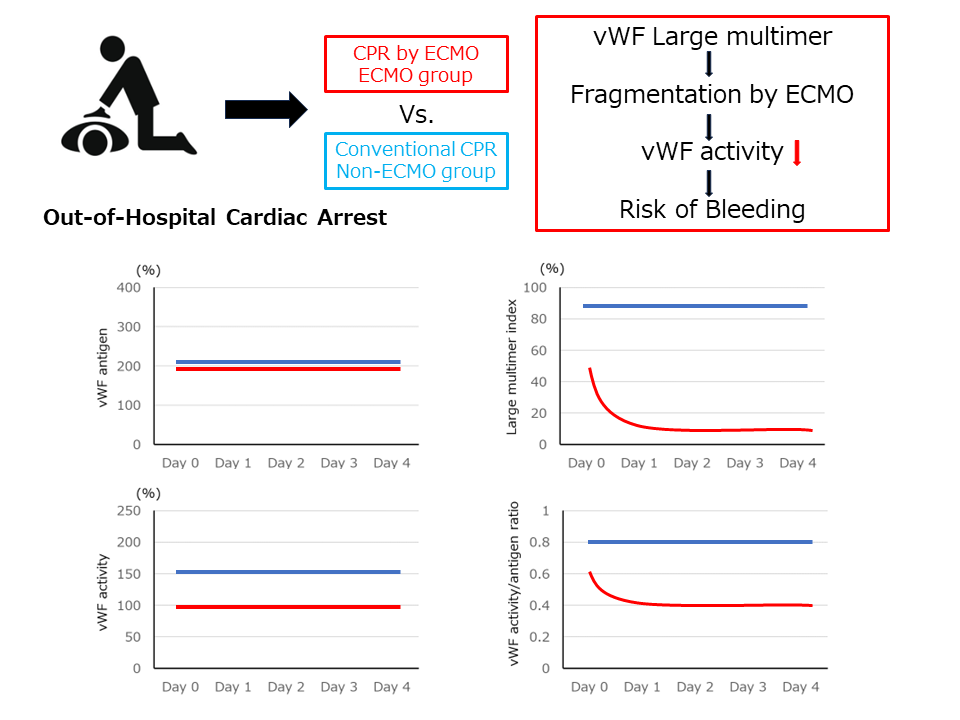

Supplement: Supplementary file 1 — Supplementary Material 1 [file 12959_2024_617_MOESM1_ESM.tif]

## Patient 1

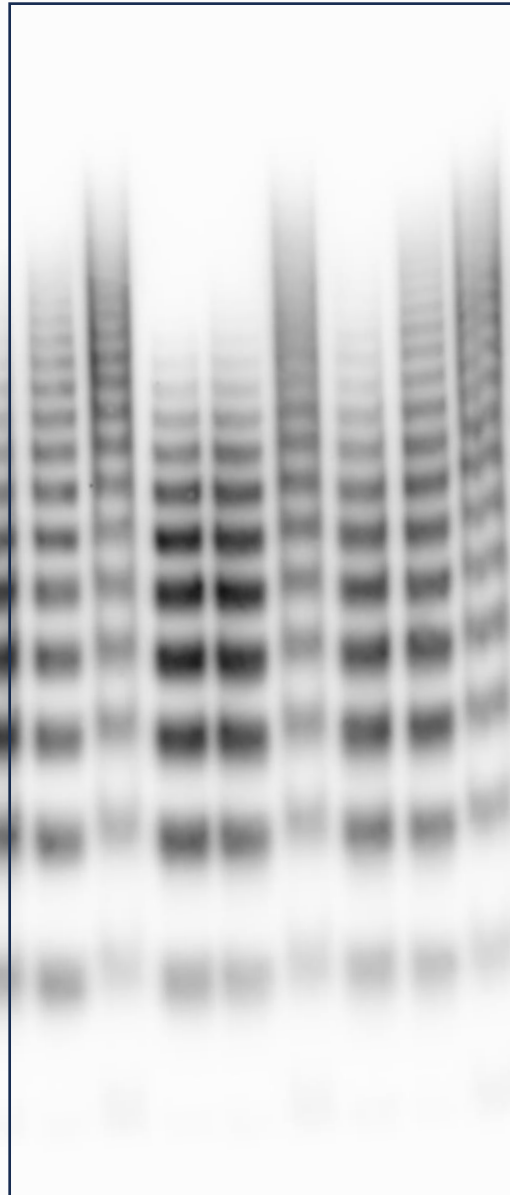

## Patient 2 Day 1 and Day 2

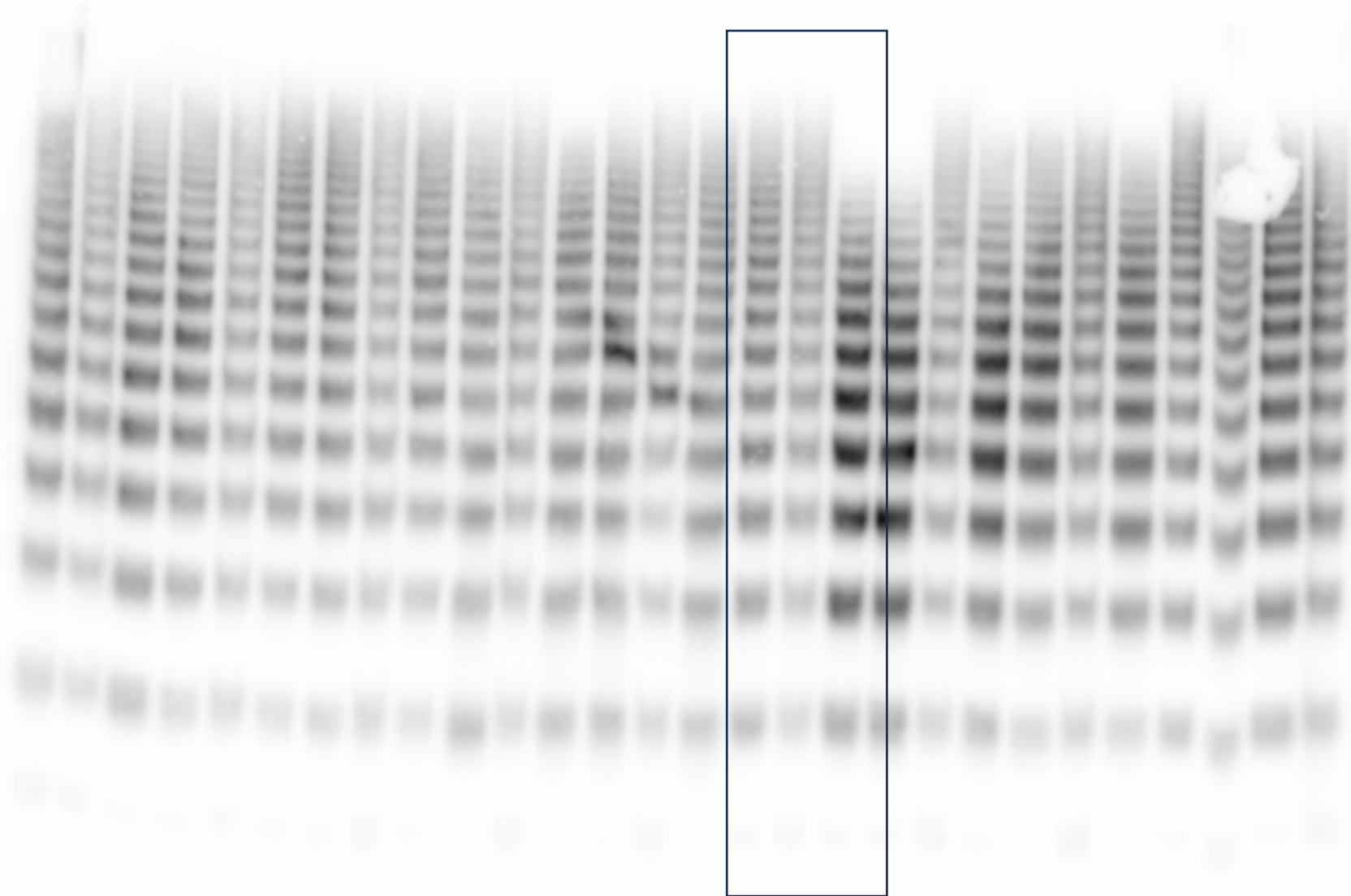

## Patient 2 Day 3 and Day 5

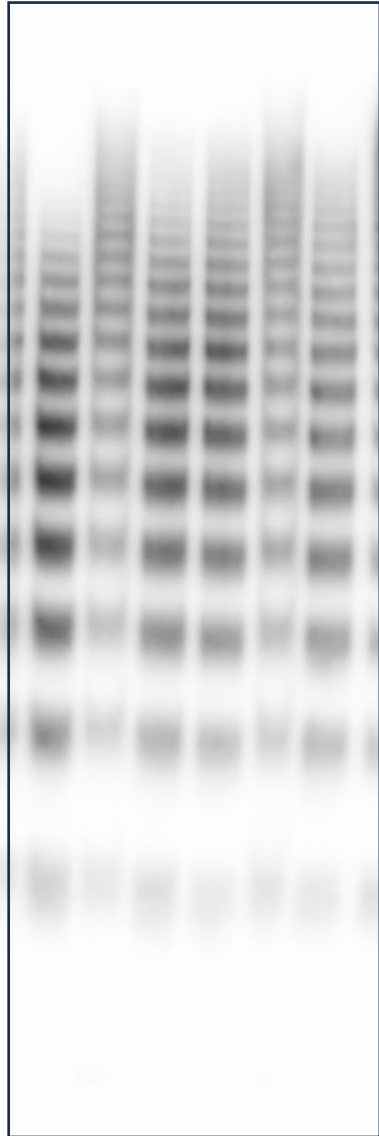

## Patient 3

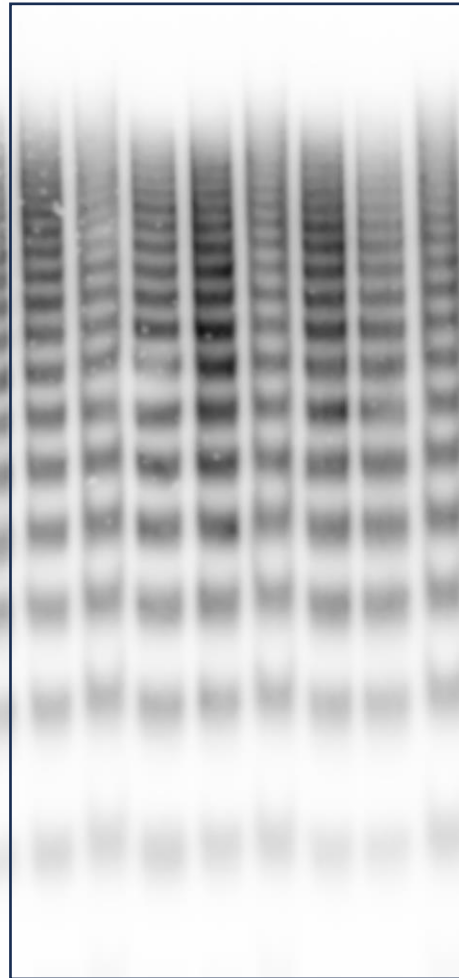

Supplement: Supplementary file 2 — Supplementary Material 2 [file 12959_2024_617_MOESM2_ESM.pdf]
